# SyNC as a cause of stroke: refining and expanding the concept and the name

**DOI:** 10.3389/fneur.2025.1643988

**Published:** 2025-08-01

**Authors:** Radostina Iordanova, Jeffery L. Saver

**Affiliations:** Los Angeles Medical Center, University of California, Los Angeles, Los Angeles, CA, United States

**Keywords:** stroke, atherosclerosis, carotid artery, Large Artery Atherosclerosis, cervical carotid artery disease

## Introduction

Recently, artery-to-artery embolism from a mild (1–49%) internal carotid artery atherosclerotic stenosis has been recognized as a common cause of ischemic stroke in patients whose events are otherwise cryptogenic (1, 2). The numbers tell the tale. In cryptogenic stroke patients, mildly stenosing plaques are present more often in the internal carotid artery that is ipsilateral, rather than contralateral, to the index infarct (3, 4). Similarly, in cryptogenic stroke patients, mildly stenosing plaques are present more often in the ipsilateral internal carotid artery than in patients with known cause stroke. In a recent meta-analysis of 16 studies enrolling 1,406 cryptogenic ischemic stroke patients, mildly stenosing internal carotid plaques with high risk features (intraplaque hemorrhage, thickness ≥3 mm, ulceration, or hypodensity) were present in 31% of ipsilateral compared with 14% of contralateral internal carotid arteries (5). Applying Bayes theorem to these contrasting rates indicates that artery-to-artery embolism from arising from mildly stenosing internal carotid stenosis is the cause of about 20% of otherwise cryptogenic anterior circulation ischemic strokes and about 4% of all ischemic strokes (6, 7).

To denote this important pathophysiologic entity, two acronyms have been advanced: SYmptomatic Non-stenosing Carotid (SyNC) disease and non-obstructive carotid atherosclerosis (NOCA), with SyNC far more widely adopted (8, 9). We will discuss these liabilities in relation to SyNC though they are equally applicable to NOCA.

## Limitations of the current appellation

The SyNC acronym incorrectly characterizes the vessel lesion as non-stenosing. The degrees of artery steno-occlusion due to atherosclerosis encompasses five broad ranges: (1) complete occlusion (100%), (2) severe stenosis (70–99%), moderately severe stenosis (50–69%), mild stenosis (1–49%), and non-stenosing (0%) (10). The 0%, non-stenosing category exists because atherosclerosis can often induce expansive vessel remodeling so that the lumen is fully preserved despite the presence of atherosclerosis (11, 12). The term “non-stenosing” in the SyNC acronym therefore formally indicates that atherosclerotic lesions that produce 0% vessel stenosis are the stroke cause. But studies of this entity have overwhelmingly used 1–49% stenosis, not 0% stenosis. as a defining feature of the culprit plaque. Accordingly, the causal lesion is best denominated as “mildly stenosing” rather than “non-stenosing.”

Another limitation is that the SyNC acronym unduly confines this pathophysiologic entity to the internal carotid artery. Atherosclerosis arises at many additional sites in the aorto-cervico-cerebral tree including the thoracic aorta, common carotid artery origin, vertebral artery origin, intracranial vertebral artery, basilar artery, posterior cerebral artery, anterior cerebral artery, and middle cerebral artery (13) Atherosclerotic lesions provoking thrombosis and artery-to-artery embolism occur at each of these locations. Consequently, the vascular lesion location for this entity is best denominated much more broadly than simply “carotid.”

There are two potential approaches to correcting the infelicities of the current word components of the SyNC acronym: (1) change the words and the acronym; or (2) change the words but select new ones that permit retention of the current acronym. We have considered a variety of new, more accurate acronyms, including: MInimally Stenotic Symptomatic Atherosclerotic Plaque (MISSAP), Minimally Obstructive Atherosclerotic Plaque (MOAP), and Large Artery Atherosclerosis—Minimally Stenosing (LAA-MS). However, the SyNC rubric is now so well-established in the literature that wide uptake of a new acronym is unlikely. Accordingly, we propose a change in its component words, to: SYmptomatic, Non-severe, Cervicocerebral (SyNC) disease. “Non-severe encompasses the full range of causative stenoses from 0 to 49% rather than only the 0% captured by “non-stenosing.” “Cervicocerebral” captures the great preponderance of vessel sites at which this entity appears, rather than the limited coverage of “carotid.” The term cervicocerebral does have the drawback of not encompassing salient thoracic vascular sites—the aorta and the common carotid and vertebral artery origins. However, adding “thoraco” to “cervicocerebral” yields an unwieldy term difficult to remember. Since specification of the arterial location of the causative plaque in individual patients is desirable, we suggest appending a location letter or letters to the acronym to convey this information: e.g., SyNC-C (Symptomatic Non-Severe Cervicocerebral Plaque—Carotid), SyNC-V (Symptomatic Non-Severe Cervicocerebral Plaque—Vertebral), and SyNC-M (Symptomatic Non-Severe Cervicocerebral Plaque—Middle Cerebral).

## Discussion

In summary, specifying the location of the atherosclerotic plaque and changing the acronym from non-stenosing to non-severe allows accurate description of stroke etiology. Precision in cerebrovascular pathophysiologic diagnosis and localization is a hallmark of vascular neurology. Correction and scope expansion of the SyNC acronym will further this core value of neurovascular research and clinical care.
